# Altered Gene Expression in Schizophrenia: Findings from Transcriptional Signatures in Fibroblasts and Blood

**DOI:** 10.1371/journal.pone.0116686

**Published:** 2015-02-06

**Authors:** Nadia Cattane, Alessandra Minelli, Elena Milanesi, Carlo Maj, Stefano Bignotti, Marco Bortolomasi, Luisella Bocchio Chiavetto, Massimo Gennarelli

**Affiliations:** 1 Department of Molecular and Translational Medicine, Biology and Genetic Division, University of Brescia, Brescia, Italy; 2 Genetics Unit, IRCCS Istituto Centro San Giovanni di Dio Fatebenefratelli, Brescia, Italy; 3 Psychiatric Unit, IRCCS Istituto Centro San Giovanni di Dio Fatebenefratelli, Brescia, Italy; 4 Psychiatric Hospital “Villa Santa Chiara”, Verona, Italy; 5 Faculty of Psychology, eCampus University, Novedrate, Como, Italy

## Abstract

**Background:**

Whole-genome expression studies in the peripheral tissues of patients affected by schizophrenia (SCZ) can provide new insight into the molecular basis of the disorder and innovative biomarkers that may be of great utility in clinical practice. Recent evidence suggests that skin fibroblasts could represent a non-neural peripheral model useful for investigating molecular alterations in psychiatric disorders.

**Methods:**

A microarray expression study was conducted comparing skin fibroblast transcriptomic profiles from 20 SCZ patients and 20 controls. All genes strongly differentially expressed were validated by real-time quantitative PCR (RT-qPCR) in fibroblasts and analyzed in a sample of peripheral blood cell (PBC) RNA from patients (n = 25) and controls (n = 22). To evaluate the specificity for SCZ, alterations in gene expression were tested in additional samples of fibroblasts and PBCs RNA from Major Depressive Disorder (MDD) (n = 16; n = 21, respectively) and Bipolar Disorder (BD) patients (n = 15; n = 20, respectively).

**Results:**

Six genes (JUN, HIST2H2BE, FOSB, FOS, EGR1, TCF4) were significantly upregulated in SCZ compared to control fibroblasts. In blood, an increase in expression levels was confirmed only for EGR1, whereas JUN was downregulated; no significant differences were observed for the other genes. EGR1 upregulation was specific for SCZ compared to MDD and BD.

**Conclusions:**

Our study reports the upregulation of JUN, HIST2H2BE, FOSB, FOS, EGR1 and TCF4 in the fibroblasts of SCZ patients. A significant alteration in EGR1 expression is also present in SCZ PBCs compared to controls and to MDD and BD patients, suggesting that this gene could be a specific biomarker helpful in the differential diagnosis of major psychoses.

## Introduction

Schizophrenia (SCZ) is a severe multifactorial disorder resulting from both genetic and environmental causes, with a lifetime risk of approximately 1% in the general population [1]. Despite the high heritability (70–90%) demonstrated by family, twin and adoption studies, its etiology remains poorly understood [2,3]. Nonetheless, converging results, ranging from different experimental approaches including animal model studies, human genetic and post-mortem brains expression data, have evidenced SCZ-related alterations of the mechanisms regulating brain neurodevelopment and neuroplasticity, dopamine and glutamate neurotransmission, neuronal myelination, the immune response system, cell adhesion and G-protein coupled or cAMP-mediated signalling [4,5,6,7,8]. According to a study of convergent functional genomics [4], among the most promising candidate genes known to play a role in the disorder are disrupted-in schizophrenia (DISC1), transcription factor 4 (TCF4), myelin basic protein (MBP) and heat-shock 70-kDa protein 1B (HSPA1B) [9,10,11,12,13].

Several lines of evidence demonstrate the usefulness of whole-genome expression studies in the identification of molecular alterations in psychiatric disorders. In this regard, an overlap has been observed between gene expression profiles in blood cells and in post-mortem brains [14,15,16], supporting the hypothesis that studies in peripheral tissues may provide new information on SCZ pathogenesis and innovative biomarkers for the diagnostic assessment and personalization of treatment. However, overall, expression studies on post-mortem brains and blood in psychiatric disorders have shown contrasting findings [15,17] and a high rate of heterogeneity that can be attributed to many sources, such as differences in sample preparation, the choice of brain region examined and other confounding factors including age, diet, smoking habit and medications [18]. These problems might be partially overcome using other peripheral tissues such as skin fibroblasts, as recently demonstrated for neurodegenerative and psychiatric disorders [19,20,21,22,23]. Indeed, fibroblasts show expression profiles that are similar to brain tissues [24], are relatively easy to obtain from patients through skin biopsy, may be maintained in culture in a controlled, reproducible environment and may also be treated with hormones and psychotropic drugs [19,20,24,25].

On this basis, the aim of our study was to identify SCZ-associated transcriptomic signatures in fibroblasts to acquire new information on the pathogenesis of the disorder and to identify new potential biomarkers for the diagnostic assessment. The experimental plan entailed the following step-by-step procedure: first, we performed a microarray gene expression study in a sample of fibroblasts from SCZ patients and controls followed by validation using real-time quantitative PCR (RT-qPCR) of the identified differentially expressed genes; second, we analyzed the mRNA expression alterations observed in fibroblasts in a sample of peripheral blood cells (PBCs) from SCZ patients; third, we also assessed the specificity of the results by analyzing SCZ gene expression alterations in fibroblasts and PBCs from patients affected by major depression (MDD) and bipolar disorder (BD).

## Methods and Materials

### Patient and control samples

The study involved patients affected by SCZ (n = 37), MDD (n = 30), and BD (n = 28) who were referred to the Psychiatric Rehabilitation Unit of IRCCS Centro S. Giovanni di Dio Fatebenefratelli, Brescia, Italy and to the “Villa S. Chiara” Psychiatric Hospital, Verona, Italy. All patients were Caucasian and had Italian descent for at least two generations. There were no relatives among the study participants, and the patients fulfilled our predefined group-specific inclusion and exclusion criteria. All SCZ patients satisfied the DSM-IV criteria for SCZ. The MDD group included DSM-IV MDD patients with at least moderately severe depression and with an absence of a lifetime history of schizophrenic, schizoaffective, or bipolar disorder and personality disorders, substance abuse, alcohol abuse or dependency, obsessive compulsive disorder, or post-traumatic stress disorder as a primary diagnosis. For inclusion in the BD group, subjects had to fulfill the DSM-IV criteria for BD and must have experienced at least two previous hypomanic and two major depressive episodes, as defined in DSM-IV.

Diagnoses were confirmed using the Structured Clinical Interview for DSM-IV Axis I Disorders (SCID-I) diagnostic scale.

The control samples (n = 41) consisted of unrelated healthy volunteers who were screened for DSM-IV Axis I disorders by expert psychologists using the Mini-International Neuropsychiatric Interview (M.I.N.I.) [26]. Only healthy volunteers without a history of drug or alcohol abuse or dependence and without a personal or first-degree family history of psychiatric disorders were enrolled in the study.

Our research project was approved by the local ethics committee (CEIOC—Fatebenefratelli hospital “San Giovanni di Dio” – Brescia, Italy, 44/2001 and 39/2005), and written informed consent was obtained from the patients and controls. As indicated by our local ethics committee guidelines, clinicians evaluated the degree to which the patients could understand several aspects of the informed consent and advisory notice. In the case of patients with a compromised ability to provide authorization, informed consent was signed by the legally authorized representative.

Exclusion criteria for the patients and controls were as follows: a) mental retardation or cognitive disorder; b) serious illnesses, including hepatic, renal, gastroenterological, respiratory, cardiovascular (including ischemic heart disease), endocrinological, neurological, immunological, or hematological disease; c) uncorrected hypothyroidism or hyperthyroidism; d) age younger than 18 and greater than 70 years

In addition, we used the following as further exclusion criteria: 1) absence of metabolic disorders (e.g., diabetes); and 2) absence of specific dermal diseases (e.g., dermal cancer or psoriasis).

The demographical and clinical features of the samples are shown in **Table 1**.

**Table 1:** Demographic and clinical features of the samples.

**Characteristics**	**Controls (N = 41)**	**SCZ (N = 37)**	**MDD (N = 30)**	**BD (N = 28)**
Age (years), mean (SD)	40.54 (12.40)	38.86 (13.43)	51.40 (11.42)	49.57 (12.91)
Gender %M	48.8	62.2	26.7	39.3
Education (years), mean (SD)	15.22 (5.10)	9.83 (2.92)	9.00 (3.97)	7.50 (2.67)
% Smokers	14.6	74.5	52.9	66.7
Age of onset (years), mean (SD)		27.00 (9.46)	35.68 (14.87)	36.92 (15.73)
% comorbidity with Axis I and/or II disorders		5.4	41.4	31.3
% administration of antipsychotics*		94.3	19.2	68.8
% administration of SSRI*		17.6	69.2	53.3
% administration of SNRI*		0.0	26.9	7.7
% administration of TCA*		0.0	11.5	28.6
% administration of NaSSA*		2.9	23.1	0.0
% administration of mood stabilizers*		14.7	11.5	69.2

*the total number could exceed the number of subjects due to the presence of multiple drugs administration

### Sample Preparation

Skin biopsies (3 mm^2^) were collected under local anesthesia from the scapular region using aseptic conditions and immediately immersed in a saline solution (PBS). All primary cultures were grown in Eagle’s minimum essential medium (MEM, Invitrogen-Life Technologies, CA, USA) supplemented with 10% of foetal bovine serum (FBS), penicillin (100 U/ml), streptomycin (100 μg/ml), non-essential amino acids (1% v/v) and glutamine (1% v/v) under optimal conditions (37°C, 5% CO_2_). The medium was replaced every 3 days. When the fibroblasts reached confluence, the cells were split into larger tissue culture dishes or were frozen using 20% FBS and 10% DMSO. As suggested by Akin and collaborators, all fibroblasts were cultured until the 5^th^ passage to minimize any effects from exposure in vivo to factors such as hormones, neurotransmitters, cytokines or drugs [25].

RNA from fibroblasts was extracted using the NucleoSpin kit (Carlo Erba Reagenti, Milano, Italy) according to the manufacturer’s instructions.

Blood samples from patients and controls were obtained by venipuncture using PaxGene Tubes (Qiagen, Crawley, UK). RNA isolation was performed using the PaxGene Blood RNA Kit (Qiagen, Crawley, UK) according to the manufacturer’s protocols.

### Microarray Procedures

The concentration of total RNA was quantified using the Nanodrop 2000 (Nanodrop Technologies, Wilmington, DE, USA) by measuring the absorbance at 260 nm. Additionally, the OD260/230 and OD260/280 ratios were determined to assess RNA purity. RNA integrity was assessed with the 2100 Bioanalyzer (Agilent Technology, TX, USA) using an RNA 6000 NanoChip and expressed as RNA Integrity Number (RIN), which was considered acceptable within the range of 7–10.

To synthesize first-strand cDNA, an aliquot of 250 ng of RNA isolated from fibroblasts was reverse-transcribed with the Ambion WT Expression Kit (Invitrogen-Life Technologies, CA, USA) using random primers tagged with a T7 promoter sequence. Second-strand cDNA was amplified by T7 RNA polymerase in an in-vitro transcription (IVT) reaction, producing many copies of antisense RNA (aRNA). Subsequently, the aRNA was purified to remove unincorporated dNTPs, salts, enzymes and inorganic phosphates. In the second cycle of cDNA synthesis with random hexamers, the aRNA was reverse-transcribed into single-stranded DNA (ssDNA) with the sense orientation. Lastly, 5.5 μg of ssDNA was fragmented and labeled with biotin. The labeled samples were hybridized onto Human Gene 1.1 ST Array Strips (Affymetrix, Inc., Santa Clara, CA, USA), comprising more than 750,000 probes and representing more than 28,000 genes mapped through UniGene or via RefSeq.

The hybridization reactions, fluidics control and imaging were performed using the Affymetrix Gene Atlas instrument according to the manufacturer’s protocol.

### Microarray Data Analysis

The oligo package was used to read the CEL files and compute the log intensities of gene expression by exploiting the Robust Multi-array Average (RMA) algorithm. Linear model fitting (LmFit) and the Empirical Bayes method (eBayes) from the BioConductor [27] limma package [28] were used to test the differences in mRNA levels.

The robust empirical Bayes procedure was used to treat outliers [29]. Only genes with a median of the log_2_-transformed expression level (*ME*) higher or equal than 6 were considered for filtering variations in the probe signal due to noise [30].

The gene lists obtained were filtered using a cut off of p-value < 0.01 and a fold change (FC) of at least ±2 (**Table 2**). Although several statistical tests permit genes with arbitrarily small fold-changes to be considered statistically significant, it has become increasingly common to require that differentially expressed genes satisfy both p-value and FC criteria simultaneously [31]. The microarray data has been submitted to Gene Expression Omnibus (accession number: GSE62333).

**Table 2:** Five transcripts differentially expressed in fibroblasts from SCZ patients compared to control subjects.

**Gene**	**Gene Assignment**	**p-value**	**Fold Change**	**Median probe intensity**
JUN	NM_002228 // JUN // jun proto-oncogene // 1p32-p31	3.14×10−7	2.08	8.45
HIST2H2BE	NM_003528 // HIST2H2BE // histone cluster 2, H2be // 1q21.2	6.48×10−7	2.27	6.52
FOSB	NM_006732 // FOSB // FBJ murine osteosarcoma viral oncogene homolog B // 19q13.32	1.18×10−4	4.87	8.08
FOS	NM_005252 // FOS // FBJ murine osteosarcoma viral oncogene homolog // 14q24.3	1.60×10−4	3.89	10.20
EGR1	NM_001964 // EGR1 // early growth response 1 // 5q31.1	4.70×10−3	2.03	10.40

The list was created using a cut off of p<0.01 and FC ± 2. The genes are ordered on the basis of the best p-values.

### Reverse Transcription and RT-qPCR Confirmation

One microgram of total RNA was used for cDNA synthesis with random hexamer primers (Invitrogen-Life Technologies, CA, USA) and Superscript II Reverse Transcriptase (Invitrogen-Life Technologies, CA, USA). The expression levels of specific transcripts in fibroblasts and PBCs were determined by RT-qPCR using the StepOnePlus instrument (Applied Biosystems, Foster City, CA, USA) with the TaqMan assay (Applied Biosystems, Foster City, CA, USA). The following genes were analyzed and normalized to the level of the housekeeping gene GAPDH (glyceraldehyde-3-phosphate dehydrogenase), Hs02758991_g1: EGR1 (early growth response 1), Hs00152928_m1; FOS (FBJ murine osteosarcoma viral oncogene homolog), Hs04194186_s1; FOSB (FBJ murine osteosarcoma viral oncogene homolog B), Hs00171851_m1; HIST2H2BE (Histone Cluster 2, H2be), Hs00269023_s1; JUN (Jun proto-oncogene), Hs01103582_s1 and TCF4, Hs00162613_m1. Each sample was assayed in duplicate. The data analyses were performed using the comparative Ct method (also known as the 2^−∆∆Ct^ method) [32].

### Statistical Analysis

The demographic and clinical characteristics of our patients and controls are described in quantitative terms using the mean±standard deviation (SD). ANOVA was used to analyze the differences between groups, and the chi-squared (χ2) test was used to evaluate categorical variables. Multiple comparisons were adjusted using the Bonferroni correction. Pearson’s coefficient was used to evaluate bivariate correlations. Logistic regression was used for adjusting the analysis for possible confounding factors.

For the prediction analysis, a receiver operating characteristic (ROC) curve analysis was used to identify the cut-off score. The area under the curve (AUC) is a measure of the overall discriminative power; a value of 0.5 represents the absence of discriminative power, whereas a value of 1.0 indicates perfect discrimination. The curve shows the different probabilities of a subject to have a risk or not of developing SCZ. Moreover, we computed the positive predictive value (PPV) and negative predictive value (NPV).

All analyses were conducted using SPSS version 17.0 statistical software (SPSS Inc. Chicago, IL, USA).

## Results

A microarray gene expression analysis was performed on fibroblasts from 20 SCZ patients (mean age±SD, 44.60±12.67; 50% males) and 20 healthy subjects (mean age±SD, 48.40±12.20; 45% males) (discovery sample). The groups were homogeneous for age (F = 0.93; p = 0.34) and gender (χ2 = 0.10; p = 0.75).

Applying the criteria described above (see microarray data analysis in Methods and Materials), the microarray output showed that five genes were significantly over-expressed in the SCZ patients compared to the controls (**Table 2**): JUN, HIST2H2BE, FOSB, FOS and EGR1. All these genes were selected as candidates for validation using the RT-qPCR technique.

Moreover, we added the TCF4 gene to the list for RT-qPCR validation, because it is one of the best candidates for SCZ pathogenesis and it was differentially expressed in our microarray analysis, albeit with a modest FC (p = 0.03; FC = 1.26; ME = 9.10).

RT-qPCR validation was performed on the validation sample. The discovery sample and the validation sample were almost completely overlapping (with 2 samples from SCZ patients higher and 1 sample from a control lower in the validation sample). All the genes selected for RT-qPCR validation are expressed in the brain, though at different levels (see **Table 3**).

**Table 3:** Brain mRNA expression levels of the 6 genes analyzed by qPCR.

	**Development**	**Adult**	**Brain areas**
**TCF4**	++	++	all
**EGR1**	+	++	all
**JUN**	++	+	all
**FOS**	−	+	all
**FOSB**	−	−	all
**HIST2H2BE**	++	+	all

The data were retrieved from a public database containing transcriptome data and associated metadata for the developing and adult human brain (http://hbatlas.org/). A total of 16 brain regions were investigated: the cerebellar cortex, mediodorsal nucleus of the thalamus, striatum, amygdala, hippocampus, and 11 areas of the neocortex. ++ = high expression,+ = average expression; − = low expression.

**Figure 1:**
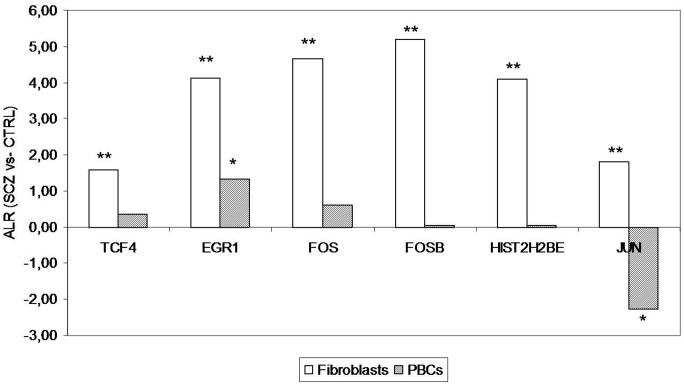
RT-qPCR mRNA expression levels from fibroblasts and PBCs expressed as Average Log2 Ratio (ALR) between the SCZ (n = 22 and n = 25, respectively) and control (n = 19 and n = 22,) samples. The bars denote the magnitude of change. All p-values were corrected for multiple comparisons by Bonferroni correction. **p≤0.001; *p≤0.05.

For the entire sample, a significant correlation was found between age and the fibroblasts mRNA levels of EGR1 (r = 0.39; p = 0.01), JUN (r = 0.36; p = 0.02), FOS (r = 0.32; p<0.05), and HIST2H2BE (r = 0.32; p = 0.04), whereas a correlation with gender was observed only for JUN (r = −0.42; p<0.01). No other significant correlations were found with sociodemographic and clinical variables. Thus, for these genes, the results were adjusted for these variables by logistic regression, with the results showing that the mRNA expression levels of all the genes were significantly different between the SCZ patients and controls (**Fig. 1**): EGR1 (F = 37.30; p = 2.5×10^−6^), FOS (F = 18.49; p = 7.8×10^−4^), FOSB (F = 33.28; p = 6.6×10^−6^), HIST2H2BE (F = 76.92; p = 6.6×10^−10^), JUN (F = 25.40; p = 7.2×10^−5^) and TCF4 (F = 14.90; p = 2.4×10^−4^). All p-values were corrected by Bonferroni correction for all comparisons (n = 6).

We subsequently performed an RT-qPCR analysis of the six genes using PBC RNA from 25 SCZ patients (35.84±11.75; 60% males) and 22 controls (32.91±6.61; 50% males). The patients and controls were homogeneous for age (F = 1.07; p = 0.31) and gender (χ2 = 0.47, p = 0.49). For the entire sample, a significant correlation was found between age and blood mRNA levels of EGR1 (r = −0.30; p = 0.04) and FOS (r = 0.40; p<0.01). No other significant correlations were found with sociodemographic and clinical variables. Consequently, for these genes, a logistic regression was performed adjusting for age as a covariate and the results showed that only EGR1 (F = 40.61; p = 6.0×10^−7^) and JUN (F = 14.33; p = 2.7×10^−3^) mRNAs were differentially expressed (**Fig. 1**). In particular, the PBC level of EGR1 mRNA was upregulated in the SCZ patients, as in fibroblasts, whereas the mRNA level of JUN was downregulated. The p-values were adjusted for multiple comparisons by Bonferroni correction. In general, all the genes analyzed were less expressed in PBCs than in fibroblasts (with a mean Ct increase of 3.58 cycles)

Because EGR1 was the only gene showing the same over-expression in both SCZ fibroblasts and PBCs, its mRNA levels were also evaluated in the fibroblasts and PBCs from MDD and BD patients to determine whether EGR1 could be a specific biomarker for SCZ or is generally involved in mental disorders.

**Figure 2:**
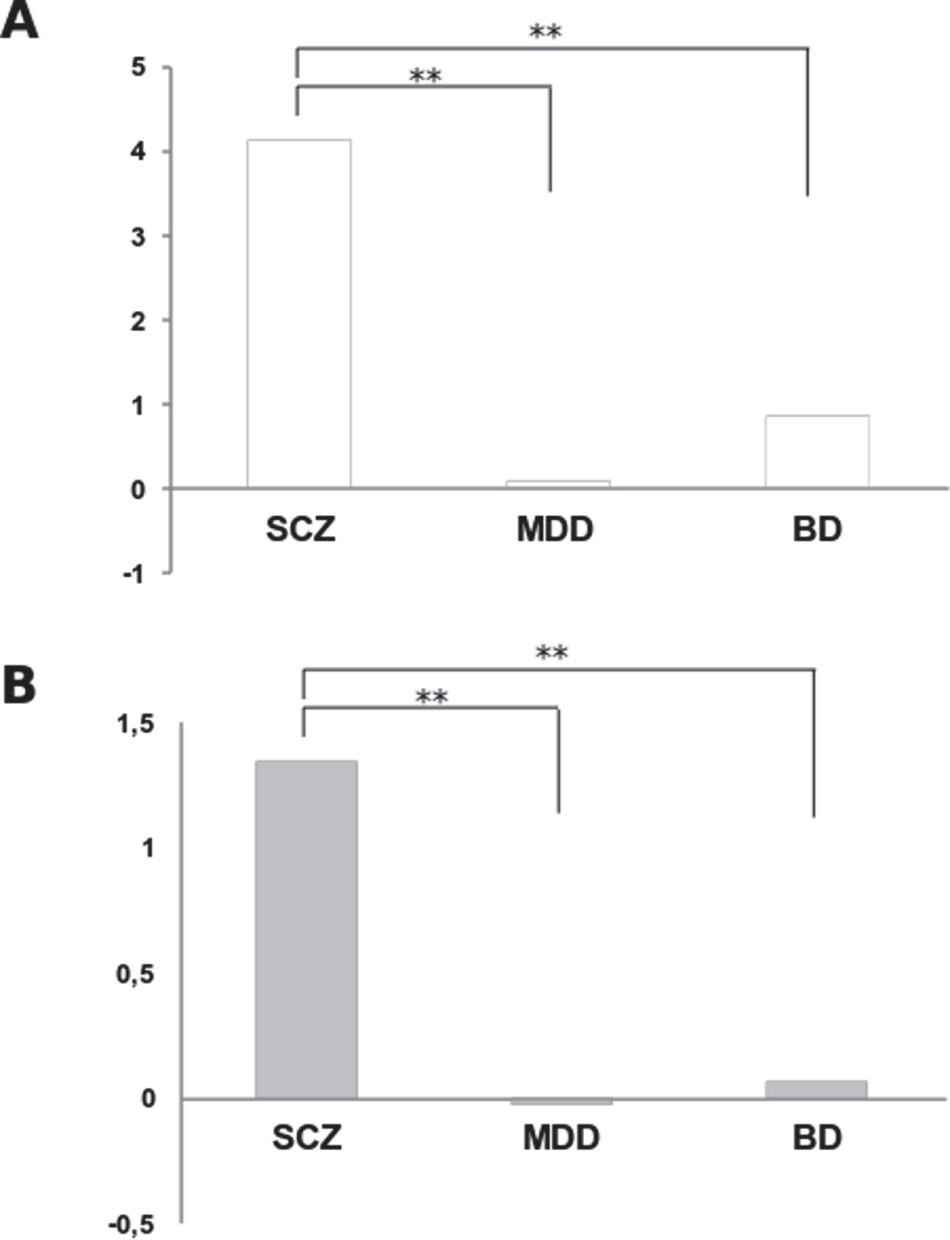
RT-qPCR of EGR1 mRNA expression levels in fibroblasts (A) and PBCs (B) from patients affected by SCZ (n = 22 and n = 25, respectively), MDD (n = 16 and n = 21) and BD (n = 15 and n = 20). The results are expressed as Average Log2 Ratio (ALR) between the SCZ, MDD and BD groups versus the control samples. The bars denote the magnitude of change. All p-values were corrected for multiple comparisons by Bonferroni correction. **p≤0.001.

A TaqMan RT-qPCR analysis was performed on fibroblast RNA from 15 BD (45.13±13.00; 26.7% males) and 16 MDD patients (49.56±11.33; 25% males). The groups were homogeneous for age (F = 1.03; p = 0.39) and gender (χ2 = 4.97, p = 0.17). In the entire sample, significant correlations were found between the fibroblast mRNA level and age (r = 0.30; p = 0.01). The results showed that the mRNA expression level of EGR1 was significantly different between the groups (F = 18.43; p = 3.0×10^−10^). Pairwise comparisons showed significant differences between the SCZ group and the MDD, BD and control groups (p = 1.8×10^−7^, p = 1.0×10^−5^ and p = 2.7×10^−8^, respectively), indicating that EGR1 mRNA level was upregulated in SCZ fibroblasts relative to all the other groups (**Fig. 2A**). All the p-values were adjusted using Bonferroni correction.

Subsequently, an RT-qPCR analysis was performed on PBC RNA from 20 BD patients in a depressed state (54.35±10.52; 45% males) and 21 MDD subjects (53.52±11.60; 23.8% males). The four groups (CTRL, SCZ, MDD and BD) were homogeneous for gender (χ2 = 6.29, p = 0.10) but not for age (F = 26.04; p = 5.26×10^−12^) and a significant correlation was found with mRNA levels (r = 0.21; p<0.05). All the results were adjusted for age by logistic regression. The data obtained showed that the mRNA expression level of EGR1 was significantly different between the groups (F = 20.39; p = 5.42×10^−10^). Pairwise comparisons showed significant differences only between the SCZ group versus the MDD, BD and controls groups (p = 5.52×10^−7^, p = 5.35×10^−6^ and p = 1.71×10^−8^, respectively), indicating that EGR1 mRNA level was upregulated only in SCZ PBCs (**Fig. 2B**). All p-values were adjusted using Bonferroni correction.

**Figure 3:**
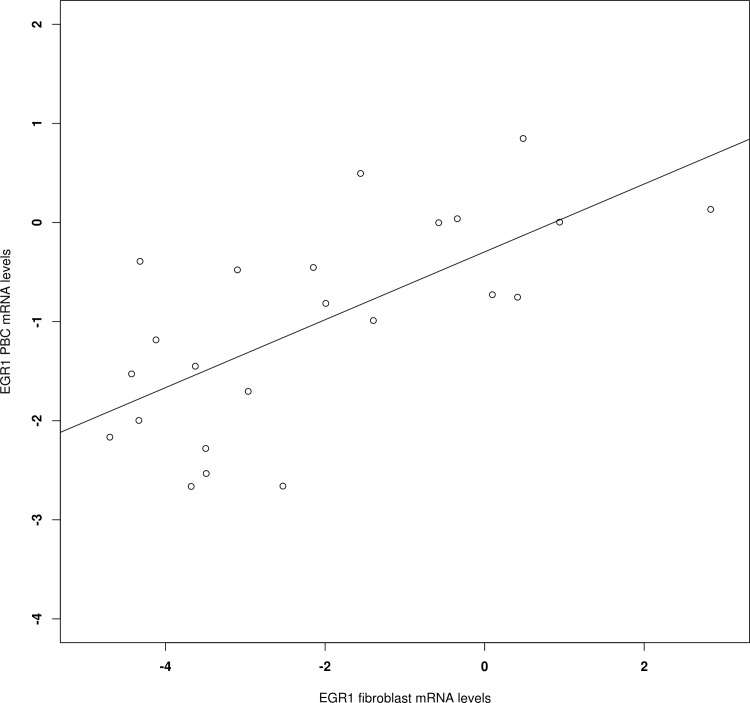
Correlation between the EGR1 mRNA levels in fibroblasts and PBCs from 24 patients for whom both tissue samples were available. The mRNA levels are expressed as ΔΔCt.

We lastly performed a correlation analysis between the fibroblasts and PBCs mRNA levels in a subgroup of 24 patients (10 SCZ, 7 MDD and 7 BD) for whom both tissues were available. The results revealed a significant correlation for EGR1 (r = 0.69; p = 1.82×10^−4^; **Fig. 3**).

With regard to the fibroblast EGR1 level, an ROC curve for predicting response showed an AUC = 0.85. In our model, the best fitting decision rule (referred to as “prediction”) revealed a sensitivity of 100%, a specificity of 78.95%, a PPV of 84.6% and an NPV of 100%. For the EGR1 PBC mRNA level, the ROC curve showed an AUC = 0.87, a sensitivity of 84.0%, a specificity of 90.9%, a PPV of 91.3% and an NPV of 83.3%.

## Discussion

Our results indicate the over-expression of JUN, HIST2H2BE, FOS, FOSB, EGR1 and TCF4 mRNA levels in the fibroblasts of SCZ patients. All these genes are expressed in the brain (see hbaltas.org; **Table 3**), albeit at different levels, and have been previously associated with SCZ [4,33,34,35,36]. The RT-qPCR results for blood RNA were in agreement with the data for fibroblasts only for EGR1, whereas the direction of expression was opposite for JUN; no significant differences were found for the other genes. Furthermore, the data showed that EGR1 over-expression in PBCs was specific for SCZ because it was not observed in BD and MDD patients.

JUN, FOS, FOSB and EGR1 are classified as immediate early genes (IEGs) because they are rapidly expressed in direct response to different stimuli. IEGs are known to encode not only transcription factors, but also a much wider variety of proteins including signaling molecules, growth factors and cytoskeletal proteins. Alterations in the expression of IEGs have been linked to stress, neurodegeneration and neuronal cell death as well as to the effects of drugs or drug of abuse administration [37,38]. Finally, recent evidence implicates IEGs in the initiation or amplification of aberrant signaling in connection with neurodegenerative processes and psychiatric disorders [39,40].

Overall, the gene expression data from human post-mortem brains and peripheral blood reporting an upregulation of JUN, FOS, FOSB in SCZ are in agreement with our results for fibroblasts [33,34,35,41].

EGR1 is a zinc finger transcription factor that was originally identified as an early response gene involved in cell proliferation, differentiation synaptic plasticity [42,43]. Recent studies have reported that an increase in EGR1 expression in the brain is associated with the formation of emotional memory [44].

Previous studies on EGR1 expression in blood are in agreement with our data [35,41,45]. In particular, increased mRNA levels were observed in the blood of SCZ patients in high delusion states [35] and in SCZ leukocytes [41]. However, the results for the brains are more conflicting: higher EGR1 levels were found in the hippocampus of an SCZ mouse model brain [35,45] whereas Yamada and colleagues [46] found reduced EGR1 transcriptional levels in the dorsolateral prefrontal cortex of the post-mortem brains from SCZ patients. Perez-Santiago and collaborators [47] did not find any differences in the expression levels of EGR1 in post-mortem human prefrontal cortex samples from SCZ patients. Moreover, it was shown that antipsychotic treatments increased EGR1 expression in the striatum of rat brains [48].

TCF4 is a transcription factor that is widely expressed in the brain, where it plays a pivotal role in neurodevelopment. Cognitive impairments and deficits in pre-pulse inhibition, such as those observed in SCZ, were found in mice over-expressing TCF4 in the forebrain [49]. These studies indicated that TCF4 may be involved in mental disorder pathogenesis and several lines of evidence with different experimental approaches (e.g., post-mortem brains, animal models) have reported alterations in TCF4 expression in SCZ [4,10,50]. Indeed, TCF4 was one of the first genes to reach genome-wide significance in large-scale genetic association studies of SCZ [51] and different single-nucleotide polymorphisms (SNPs) have been associated with the disorder in independent studies [52,53,54]. This gene is also the target of several microRNAs, including the mir-137, which was implicated in SCZ etiology in GWAS studies [55]. In particular, the SCZ-associated rs1625579 SNP in miR-137 was correlated with the decreased expression of this microRNA in the dorsolateral prefrontal cortex and a consequent increase in TCF4 levels [56]. Furthermore, increased levels of TCF4 mRNA were observed in IPSC-derived neurons obtained from SCZ patient fibroblasts [1]. Data on blood cell RNA levels are more contrasting and might be influenced by the psychotic state and symptomatology [35,50,52].

HIST2H2BE encodes a member of the histone H2B family, which is mainly involved in immune response, cell growth, DNA damage, signal transduction and inflammatory reactions. Several studies report alterations in histones methylation at specific genes in the post-mortem brain of SCZ subjects, often in conjunction with changes in the levels of the corresponding RNAs [57,58]. The typical working hypothesis suggests that a change in promoter DNA methylation or histone modification can induce lasting and stable changes in gene expression, and these alterations have therefore been implicated in promoting the adaptive behavioral and neuronal changes in SCZ [58]. In a recent article, Mattheisen and colleagues tested all common variants in the synaptic vesicle glycoprotein 2A gene (SV2A) region for an association with SCZ. They found that the rs15931 SNP, which covers the genomic region proximal to SV2A, is located in the 3′UTR of HIST2H2BE, showing a possible involvement of HIST2H2BE in SCZ [36].

The results obtained in SCZ fibroblasts support previous findings involving IEGs and TCF4 in SCZ and suggest an alteration in HIST2H2BE gene expression. Moreover, our data demonstrate the usefulness of skin fibroblasts as a peripheral model for studying psychiatric disorders.

Indeed, fibroblasts are a promising model for studying central nervous system (CNS) disorders [59] because these cells share certain features with cells of neuro-ectodermal origin [60] and can be transformed into neurons [1,61,62].

Recent evidence has indicated that fibroblasts are a convenient and useful tool for investigating several processes in SCZ, such as the immune response or cytokine stimulation [63,64,65] apoptotic susceptibility [23], tyrosine transport across plasma membranes in response to antipsychotic drug treatment [66] or regulation of gene expression in patients deficient in glutathione synthesis [67].

Skin fibroblast cultures are easy to prepare and maintain without transformation, and many possible confounding factors often associated with patient peripheral tissues, such as diet, lifestyles, smoking habit and drug treatment may be virtually eliminated after several cycles of cell division [59].

In this study, we analyzed genes differentially expressed in SCZ fibroblasts and SCZ PBCs, reporting a parallel over-expression of EGR1. In contrast, JUN mRNA levels were decreased in PBCs from SCZ patients, whereas the expression of the other evaluated genes was not altered in these cells. The lack of data replication for PBCs (except for EGR1) might be attributed to the lower expression level (with respect to fibroblasts) of the candidate gene in blood. In fact, comparing the RT-qPCR data for PBCs and fibroblasts from the control sample, we observed a 3.58 mean increase in the number of cycles (mean Ct) that corresponded to a decrease in the mRNA concentrations.

The cellular component of peripheral blood is largely composed of immune cells, such as leukocytes, wich express a wide repertoire of cytokines and their receptors, neuroendocrine hormones and neuropeptides, neurotrophic factors such as brain-derived neurotrophic factor or dopamine and serotonin receptors. For these reasons, this tissue might be more helpful for testing hypotheses regarding the relationship between immune response alterations and psychiatric disorders [59].

However, the identification of blood signatures associated with diagnoses in psychiatric disorders might be more useful in the development of innovative biomarkers that are easily applicable in clinical practice. Furthermore, mRNA measurements using blood are less invasive, less expensive and more rapid than those using fibroblasts.

Thus, our data on EGR1 levels in blood are particularly interesting because they reveal a putative specific marker able to differentiate SCZ from other major psychosis patients.

Nevertheless, fibroblasts can be more advantageous for discovering mental disorder etiological mechanisms because they appear to be more similar to neurons and less affected by environmental confounders (such as diet, medication and smoking). Finally, cultured fibroblasts may be treated with psychotropic drugs to observe molecular and signaling modifications induced by the treatment [24]. In this regard, it might be interesting to observe whether antipsychotic treatments are able to “normalize” the expression alterations observed in this study.

In conclusion, this study shows the upregulation of JUN, HIST2H2BE, FOS, FOSB, EGR1 and TCF4 in SCZ fibroblasts, confirming the role of these genes in this disorder. Furthermore, our results identify EGR1 as a specific biomarker of SCZ, which might be helpful for the differential diagnosis of major psychoses.

## References

[1] Brennand KJ, Simone A, Tran N, Gage FH (2012) Modeling psychiatric disorders at the cellular and network levels. Mol Psychiatry 17: 1239–1253. 10.1038/mp.2012.20 22472874 PMC3465628

[2] Mowry BJ, Gratten J (2013) The emerging spectrum of allelic variation in schizophrenia: current evidence and strategies for the identification and functional characterization of common and rare variants. Mol Psychiatry 18: 38–52. 10.1038/mp.2012.34 22547114

[3] Girard SL, Dion PA, Rouleau GA (2012) Schizophrenia genetics: putting all the pieces together. Curr Neurol Neurosci Rep 12: 261–266. 10.1007/s11910-012-0266-7 22456906

[4] Ayalew M, Le-Niculescu H, Levey DF, Jain N, Changala B, et al. (2012) Convergent functional genomics of schizophrenia: from comprehensive understanding to genetic risk prediction. Mol Psychiatry 17: 887–905. 10.1038/mp.2012.37 22584867 PMC3427857

[5] Hall J, Trent S, Thomas KL, O’Donovan MC, Owen MJ (2014) Genetic Risk for Schizophrenia: Convergence on Synaptic Pathways Involved in Plasticity. Biol Psychiatry. 10.1016/j.biopsych.2014.07.011 25152434

[6] Tomasik J, Rahmoune H, Guest PC, Bahn S (2014) Neuroimmune biomarkers in schizophrenia. Schizophr Res. 10.1016/j.schres.2014.07.025 25124519

[7] Pitman KA, Puil E, Borgland SL (2014) GABA modulation of dopamine release in the nucleus accumbens core. Eur J Neurosci. 10.1111/ejn.12733 25229321

[8] Steullet P, Cabungcal JH, Monin A, Dwir D, O’Donnell P, et al. (2014) Redox dysregulation, neuroinflammation, and NMDA receptor hypofunction: A “central hub” in schizophrenia pathophysiology? Schizophr Res. 10.1016/j.schres.2014.06.021 PMC4282982 25000913

[9] Quednow BB, Brzozka MM, Rossner MJ (2014) Transcription factor 4 (TCF4) and schizophrenia: integrating the animal and the human perspective. Cell Mol Life Sci 71: 2815–2835. 10.1007/s00018-013-1553-4 24413739 PMC11113759

[10] Forrest MP, Hill MJ, Quantock AJ, Martin-Rendon E, Blake DJ (2014) The emerging roles of TCF4 in disease and development. Trends Mol Med 20: 322–331. 10.1016/j.molmed.2014.01.010 24594265

[11] Duff BJ, Macritchie KA, Moorhead TW, Lawrie SM, Blackwood DH (2013) Human brain imaging studies of DISC1 in schizophrenia, bipolar disorder and depression: a systematic review. Schizophr Res 147: 1–13. 10.1016/j.schres.2013.03.015 23602339

[12] Matthews PR, Eastwood SL, Harrison PJ (2012) Reduced myelin basic protein and actin-related gene expression in visual cortex in schizophrenia. PLoS One 7: e38211 10.1371/journal.pone.0038211 22675524 PMC3365879

[13] Pae CU, Drago A, Kim JJ, Mandelli L, De Ronchi D, et al. (2009) The impact of heat shock protein 70 gene variations on clinical presentation and outcome in schizophrenic inpatients. Neuropsychobiology 59: 135–141. 10.1159/000218075 19439993

[14] Rollins B, Martin MV, Morgan L, Vawter MP (2010) Analysis of whole genome biomarker expression in blood and brain. Am J Med Genet B Neuropsychiatr Genet 153B: 919–936. 10.1002/ajmg.b.31062 20127885 PMC3098564

[15] Sullivan PF, Fan C, Perou CM (2006) Evaluating the comparability of gene expression in blood and brain. Am J Med Genet B Neuropsychiatr Genet 141B: 261–268. 16526044 10.1002/ajmg.b.30272

[16] Tylee DS, Kawaguchi DM, Glatt SJ (2013) On the outside, looking in: a review and evaluation of the comparability of blood and brain “-omes". Am J Med Genet B Neuropsychiatr Genet 162B: 595–603. 10.1002/ajmg.b.32150 24132893

[17] Mistry M, Gillis J, Pavlidis P (2013) Genome-wide expression profiling of schizophrenia using a large combined cohort. Mol Psychiatry 18: 215–225. 10.1038/mp.2011.172 22212594 PMC3323740

[18] Kumarasinghe N, Tooney PA, Schall U (2012) Finding the needle in the haystack: a review of microarray gene expression research into schizophrenia. Aust N Z J Psychiatry 46: 598–610. 10.1177/0004867412442405 22441207

[19] Auburger G, Klinkenberg M, Drost J, Marcus K, Morales-Gordo B, et al. (2012) Primary skin fibroblasts as a model of Parkinson’s disease. Mol Neurobiol 46: 20–27. 10.1007/s12035-012-8245-1 22350618 PMC3443476

[20] Garbett KA, Vereczkei A, Kalman S, Brown JA, Taylor WD, et al. (2014) Coordinated Messenger RNA/MicroRNA Changes in Fibroblasts of Patients with Major Depression. Biol Psychiatry. 10.1016/j.biopsych.2014.05.015 25016317 PMC4254393

[21] Matigian NA, McCurdy RD, Feron F, Perry C, Smith H, et al. (2008) Fibroblast and lymphoblast gene expression profiles in schizophrenia: are non-neural cells informative? PLoS One 3: e2412 10.1371/journal.pone.0002412 18545665 PMC2398775

[22] Wang L, Lockstone HE, Guest PC, Levin Y, Palotas A, et al. (2010) Expression profiling of fibroblasts identifies cell cycle abnormalities in schizophrenia. J Proteome Res 9: 521–527. 10.1021/pr900867x 19916557

[23] Gasso P, Mas S, Molina O, Lafuente A, Bernardo M, et al. (2013) Increased susceptibility to apoptosis in cultured fibroblasts from antipsychotic-naive first-episode schizophrenia patients. J Psychiatr Res. 10.1016/j.jpsychires.2013.09.017 24128664

[24] Kalman S, Garbett KA, Vereczkei A, Shelton RC, Korade Z, et al. (2014) Metabolic stress-induced microRNA and mRNA expression profiles of human fibroblasts. Exp Cell Res 320: 343–353. 10.1016/j.yexcr.2013.10.019 24246224 PMC3902643

[25] Akin D, Manier DH, Sanders-Bush E, Shelton RC (2004) Decreased serotonin 5-HT2A receptor-stimulated phosphoinositide signaling in fibroblasts from melancholic depressed patients. Neuropsychopharmacology 29: 2081–2087. 15187984 10.1038/sj.npp.1300505

[26] Sheehan DV, Lecrubier Y, Sheehan KH, Amorim P, Janavs J, et al. (1998) The Mini-International Neuropsychiatric Interview (M.I.N.I.): the development and validation of a structured diagnostic psychiatric interview for DSM-IV and ICD-10. J Clin Psychiatry 59 Suppl 20: 22–33;quiz 34–57. 9881538

[27] Gentleman RC, Carey VJ, Bates DM, Bolstad B, Dettling M, et al. (2004) Bioconductor: open software development for computational biology and bioinformatics. Genome Biol 5: R80 10.1186/gb-2004-5-10-r80 15461798 PMC545600

[28] Smyth GK, Michaud J, Scott HS (2005) Use of within-array replicate spots for assessing differential expression in microarray experiments. Bioinformatics 21: 2067–2075. 15657102 10.1093/bioinformatics/bti270

[29] Phipson B, Smyth GK (2010) Permutation P-values should never be zero: calculating exact P-values when permutations are randomly drawn. Stat Appl Genet Mol Biol 9: Article39 10.2202/1544-6115.1585 21044043

[30] Kang HJ, Kawasawa YI, Cheng F, Zhu Y, Xu X, et al. (2011) Spatio-temporal transcriptome of the human brain. Nature 478: 483–489. 10.1038/nature10523 22031440 PMC3566780

[31] McCarthy DJ, Smyth GK (2009) Testing significance relative to a fold-change threshold is a TREAT. Bioinformatics 25: 765–771. 10.1093/bioinformatics/btp053 19176553 PMC2654802

[32] Schmittgen TD, Livak KJ (2008) Analyzing real-time PCR data by the comparative C(T) method. Nat Protoc 3: 1101–1108. 18546601 10.1038/nprot.2008.73

[33] Kyosseva SV (2004) Differential expression of mitogen-activated protein kinases and immediate early genes fos and jun in thalamus in schizophrenia. Prog Neuropsychopharmacol Biol Psychiatry 28: 997–1006. 15380860 10.1016/j.pnpbp.2004.05.017

[34] Todorova VK, Elbein AD, Kyosseva SV (2003) Increased expression of c-Jun transcription factor in cerebellar vermis of patients with schizophrenia. Neuropsychopharmacology 28: 1506–1514. 12799614 10.1038/sj.npp.1300211

[35] Kurian SM, Le-Niculescu H, Patel SD, Bertram D, Davis J, et al. (2011) Identification of blood biomarkers for psychosis using convergent functional genomics. Mol Psychiatry 16: 37–58. 10.1038/mp.2009.117 19935739

[36] Mattheisen M, Muhleisen TW, Strohmaier J, Treutlein J, Nenadic I, et al. (2012) Genetic variation at the synaptic vesicle gene SV2A is associated with schizophrenia. Schizophr Res 141: 262–265. 10.1016/j.schres.2012.08.027 23017826

[37] Hansson AC, Fuxe K (2008) Time-course of immediate early gene expression in hippocampal subregions of adrenalectomized rats after acute corticosterone challenge. Brain Res 1215: 1–10. 10.1016/j.brainres.2008.03.080 18485334 PMC2435408

[38] Koldamova R, Schug J, Lefterova M, Cronican AA, Fitz NF, et al. (2014) Genome-wide approaches reveal EGR1-controlled regulatory networks associated with neurodegeneration. Neurobiol Dis 63: 107–114. 10.1016/j.nbd.2013.11.005 24269917 PMC3939039

[39] Hendrickx A, Pierrot N, Tasiaux B, Schakman O, Kienlen-Campard P, et al. (2014) Epigenetic regulations of immediate early genes expression involved in memory formation by the amyloid precursor protein of Alzheimer disease. PLoS One 9: e99467 10.1371/journal.pone.0099467 24919190 PMC4053420

[40] Reul JM (2014) Making memories of stressful events: a journey along epigenetic, gene transcription, and signaling pathways. Front Psychiatry 5: 5 10.3389/fpsyt.2014.00005 24478733 PMC3897878

[41] Middleton FA, Pato CN, Gentile KL, McGann L, Brown AM, et al. (2005) Gene expression analysis of peripheral blood leukocytes from discordant sib-pairs with schizophrenia and bipolar disorder reveals points of convergence between genetic and functional genomic approaches. Am J Med Genet B Neuropsychiatr Genet 136B: 12–25. 15892139 10.1002/ajmg.b.30171

[42] Zhang L, Cho J, Ptak D, Leung YF (2013) The role of egr1 in early zebrafish retinogenesis. PLoS One 8: e56108 10.1371/journal.pone.0056108 23405257 PMC3566060

[43] Gomez Ravetti M, Rosso OA, Berretta R, Moscato P (2010) Uncovering molecular biomarkers that correlate cognitive decline with the changes of hippocampus’ gene expression profiles in Alzheimer’s disease. PLoS One 5: e10153 10.1371/journal.pone.0010153 20405009 PMC2854141

[44] Baumgartel K, Genoux D, Welzl H, Tweedie-Cullen RY, Koshibu K, et al. (2008) Control of the establishment of aversive memory by calcineurin and Zif268. Nat Neurosci 11: 572–578. 10.1038/nn.2113 18425121

[45] Le-Niculescu H, Balaraman Y, Patel S, Tan J, Sidhu K, et al. (2007) Towards understanding the schizophrenia code: an expanded convergent functional genomics approach. Am J Med Genet B Neuropsychiatr Genet 144B: 129–158. 17266109 10.1002/ajmg.b.30481

[46] Yamada K, Gerber DJ, Iwayama Y, Ohnishi T, Ohba H, et al. (2007) Genetic analysis of the calcineurin pathway identifies members of the EGR gene family, specifically EGR3, as potential susceptibility candidates in schizophrenia. Proc Natl Acad Sci U S A 104: 2815–2820. 10.1073/pnas.0610765104 17360599 PMC1815264

[47] Perez-Santiago J, Diez-Alarcia R, Callado LF, Zhang JX, Chana G, et al. (2012) A combined analysis of microarray gene expression studies of the human prefrontal cortex identifies genes implicated in schizophrenia. J Psychiatr Res 46: 1464–1474. 10.1016/j.jpsychires.2012.08.005 22954356

[48] Bruins Slot LA, Lestienne F, Grevoz-Barret C, Newman-Tancredi A, Cussac D (2009) F15063, a potential antipsychotic with dopamine D(2)/D(3) receptor antagonist and 5-HT(1A) receptor agonist properties: influence on immediate-early gene expression in rat prefrontal cortex and striatum. Eur J Pharmacol 620: 27–35. 10.1016/j.ejphar.2009.08.019 19695244

[49] Brzozka MM, Radyushkin K, Wichert SP, Ehrenreich H, Rossner MJ (2010) Cognitive and sensorimotor gating impairments in transgenic mice overexpressing the schizophrenia susceptibility gene Tcf4 in the brain. Biol Psychiatry 68: 33–40. 10.1016/j.biopsych.2010.03.015 20434134

[50] Navarrete K, Pedroso I, De Jong S, Stefansson H, Steinberg S, et al. (2013) TCF4 (e2–2; ITF2): a schizophrenia-associated gene with pleiotropic effects on human disease. Am J Med Genet B Neuropsychiatr Genet 162: 1–16. 10.1002/ajmg.b.32109 23129290

[51] Stefansson H, Ophoff RA, Steinberg S, Andreassen OA, Cichon S, et al. (2009) Common variants conferring risk of schizophrenia. Nature 460: 744–747. 10.1038/nature08186 19571808 PMC3077530

[52] Wirgenes KV, Sonderby IE, Haukvik UK, Mattingsdal M, Tesli M, et al. (2012) TCF4 sequence variants and mRNA levels are associated with neurodevelopmental characteristics in psychotic disorders. Transl Psychiatry 2: e112 10.1038/tp.2012.39 22832956 PMC3365258

[53] Steinberg S, de Jong S, Andreassen OA, Werge T, Borglum AD, et al. (2011) Common variants at VRK2 and TCF4 conferring risk of schizophrenia. Hum Mol Genet 20: 4076–4081. 10.1093/hmg/ddr325 21791550 PMC3298077

[54] Lennertz L, Quednow BB, Benninghoff J, Wagner M, Maier W, et al. (2011) Impact of TCF4 on the genetics of schizophrenia. Eur Arch Psychiatry Clin Neurosci 261 Suppl 2: S161–165. 10.1007/s00406-011-0256-9 21932083

[55] Ripke S, O’Dushlaine C, Chambert K, Moran JL, Kahler AK, et al. (2013) Genome-wide association analysis identifies 13 new risk loci for schizophrenia. Nat Genet 45: 1150–1159. 10.1038/ng.2742 23974872 PMC3827979

[56] Guella I, Sequeira A, Rollins B, Morgan L, Torri F, et al. (2013) Analysis of miR-137 expression and rs1625579 in dorsolateral prefrontal cortex. J Psychiatr Res 47: 1215–1221. 10.1016/j.jpsychires.2013.05.021 23786914 PMC3753093

[57] Akbarian S (2010) Epigenetics of schizophrenia. Curr Top Behav Neurosci 4: 611–628. 21312415 10.1007/7854_2010_38

[58] Mahgoub M, Monteggia LM (2013) Epigenetics and psychiatry. Neurotherapeutics 10: 734–741. 10.1007/s13311-013-0213-6 24092614 PMC3805856

[59] Hayashi-Takagi A, Vawter MP, Iwamoto K (2014) Peripheral biomarkers revisited: integrative profiling of peripheral samples for psychiatric research. Biol Psychiatry 75: 920–928. 10.1016/j.biopsych.2013.09.035 24286759 PMC4964959

[60] Rieske P, Krynska B, Azizi SA (2005) Human fibroblast-derived cell lines have characteristics of embryonic stem cells and cells of neuro-ectodermal origin. Differentiation 73: 474–483. 10.1038/nature10284 16351691

[61] Caiazzo M, Dell’Anno MT, Dvoretskova E, Lazarevic D, Taverna S, et al. (2011) Direct generation of functional dopaminergic neurons from mouse and human fibroblasts. Nature 476: 224–227. 21725324 10.1038/nature10284

[62] Theka I, Caiazzo M, Dvoretskova E, Leo D, Ungaro F, et al. (2013) Rapid generation of functional dopaminergic neurons from human induced pluripotent stem cells through a single-step procedure using cell lineage transcription factors. Stem Cells Transl Med 2: 473–479. 10.5966/sctm.2012-0133 23658252 PMC3673759

[63] Johansson AS, Owe-Larsson B, Asp L, Kocki T, Adler M, et al. (2013) Activation of kynurenine pathway in ex vivo fibroblasts from patients with bipolar disorder or schizophrenia: cytokine challenge increases production of 3-hydroxykynurenine. J Psychiatr Res 47: 1815–1823. 10.1016/j.jpsychires.2013.08.008 24012176

[64] Wolf J, Weinberger B, Arnold CR, Maier AB, Westendorp RG, et al. (2012) The effect of chronological age on the inflammatory response of human fibroblasts. Exp Gerontol 47: 749–753. 10.1016/j.exger.2012.07.001 22790019 PMC3427851

[65] Asp L, Johansson AS, Mann A, Owe-Larsson B, Urbanska EM, et al. (2011) Effects of pro-inflammatory cytokines on expression of kynurenine pathway enzymes in human dermal fibroblasts. J Inflamm (Lond) 8: 25 10.1186/1476-9255-8-25 21982155 PMC3204223

[66] Bongiovanni R, Leonard S, Jaskiw GE (2013) A simplified method to quantify dysregulated tyrosine transport in schizophrenia. Schizophr Res 150: 386–391. 10.1016/j.schres.2013.08.041 24051014

[67] Monin A, Baumann PS, Griffa A, Xin L, Mekle R, et al. (2014) Glutathione deficit impairs myelin maturation: relevance for white matter integrity in schizophrenia patients. Mol Psychiatry. 10.1038/mp.2014.88 25155877

